# Identification and Functional Analysis of a Novel Hydrophobic Protein VdHP1 from Verticillium dahliae

**DOI:** 10.1128/spectrum.02478-21

**Published:** 2022-04-04

**Authors:** Xiaojian Zhang, Lihong Zhao, Shichao Liu, Jinglong Zhou, Yajie Wu, Zili Feng, Yalin Zhang, Heqin Zhu, Feng Wei, Hongjie Feng

**Affiliations:** a Zhengzhou Research Base, State Key Laboratory of Cotton Biology, School of Agricultural Sciences, Zhengzhou University, Henan, Zhengzhou, China; b Western Agricultural Research Center of Chinese Academy of Agricultural Sciences, Xinjiang, Changji, China; c State Key Laboratory of Cotton Biology, Institute of Cotton Research of Chinese Academy of Agricultural Sciences, Anyang, Henan, China; USDA–San Joaquin Valley Agricultural Sciences Center

**Keywords:** *Verticillium dahliae*, hydrophobin, biological characteristics, virulence, stress response, pathogenicity

## Abstract

Verticillium dahliae could cause destructive vascular wilt disease on hundreds of plant species around the world, including cotton. In this study, we characterized the function of a hydrophobin gene *VdHP1* in pathogen development and pathogenicity. Results showed that *VdHP1* could induce cell death and activate plant immune responses. The *VdHP1* deletion mutants (*ΔVdHP1*) and the complement mutants (*C-ΔVdHP1*) were obtained by the homologous recombination method. The *VdHP1* deletion mutants exhibited increased hydrophilicity, inhibited microsclerotial formation, and reduced spore smoothness. In addition, the deletion mutants were more sensitive to NaCl, while relatively insensitive to KCl and sorbitol. Mutants also had greater resistance to Congo red, UV radiation, and high temperature, which suggested that *ΔVdHP1* strains have stronger resistance to abiotic stress in general. Different carbon source assays showed that the utilization ability of skim milk, cellulose, and starch was greatly enhanced in *ΔVdHP1*, compared with that of WT and complemented strains. Furthermore, *VdHP1* did not affect mycelium penetration on cellophane but contributed to mycelium growth on surface of the living plant cells. The pathogenicity test found that the crude toxin content, colonization, and dispersal of *ΔVdHP1* was significantly increased compared with the WT and complementary strains. In addition, cotton seedlings showed more severe wilting symptoms after inoculation with *ΔVdHP1* strains. These results suggested that the hydrophobin *VdHP1* negatively regulated the virulence of V. dahliae, and played an important role in development, adaptability, and pathogenicity in V. dahliae, which maybe provide a new viewpoint to further understand the molecular mechanisms of pathogen virulence.

**IMPORTANCE** Verticillium dahliae is a soilborne fungal pathogen that causes a destructive vascular disease on a large number of plant hosts, resulting in great threat to agricultural production. In this study, it was illustrated that the hydrophobin VdHP1 could induce cell death and activate plant immune responses. VdHP1 affected the hydrophobicity of V. dahliae, and negatively regulated the strains resistant to stress, and the utilization ability of different carbon sources. In addition, VdHP1 did not affect mycelium penetration on cellophane but contributed to mycelium growth on surface of the living plant cells. The *VdHP1* gene negatively regulated the total virulence, colonization, and dispersal of V. dahliae, with enhanced pathogenicity of mutant strains in this gene. These results suggested that the hydrophobin VdHP1 played an importance in development, adaptability, and pathogenicity in V. dahliae, and would provide a new viewpoint to further understand the molecular mechanisms of pathogen virulence.

## INTRODUCTION

Phytopathogenic fungus Verticillium dahliae can infect more than 200 species of dicotyledons, and cause a serious vascular wilt disease (1, 2). It is responsible for cotton vascular wilt diseases at any lifestage, but no effective fungicide to control Verticillium wilt is available (3, 4). Verticillium wilt has caused serious economic losses on cotton production in China (5, 6). Microsclerotia, the dormant structure, is the primary inoculum of V. dahliae (7–9). These structures can help the pathogen resist extreme temperatures, desiccation, and other environmental stresses. Once the environment is suitable, hyphae will be formed by microsclerotia of V. dahliae. The hyphae near or in contact with the roots grow and spread rapidly and enter into the host through the root tip and wounds on the root. On invasion, the fungi stay for a brief period in the cortex, before entering the xylem tissue. In this process, pathogens need to overcome tissue barriers such as the cell wall of the host plant (10). The spores of fungi are ubiquitous propagation structures that are also often the infectious agents of diseases (11). Furthermore, the spores V. dahliae could enhance the resistance to high levels of UV, desiccation, pressure, heat, and cold, for ensuring the survival of spores in the harshest conditions. It was reported that V. dahliae spores can survive at 4°C (12). Beauveria bassiana spores still germinate at 45°C (13). Spore germination then leads to filamentous growth, which would result in tissue damage to the host plants. For example, the expansion of Fusarium graminearum mycelia is the main way of invading plant tissues (14). V. dahliae has the dormant structure of microsclerotia, highly stress resistant spores, and broad host range that makes it very difficult to control. Until now, the main research has focused on the molecular mechanism of pathogenic pathogenic genes to explore virulence factors.

In terms of virulence, *VdBre1* affected the total virulence by regulating lipid metabolism and secondary metabolites in V. dahliae (15). Pyrimidine biosynthesis related gene *VdTHI20* was also necessary for pathogenicity (7). *VdCrz1* and *VdMcm1*, two transcription factors, had been reported to be essential for plant infection (16, 17). The reaction regulator *VdSsk1* was involved in stress response, melanin biosynthesis, and total virulence of V. dahliae (18). Knockout of pathogenicity-related genes *VdPR1* and *VdPR3* of V. dahliae would reduce the pathogenicity of V. dahliae (19, 20). Currently, there are two hypotheses about the pathogenic mechanism of V. dahliae (21). One hypothesis is that it is predominantly the result of vessel occlusion in the plant, whereas the other states that Verticillium wilt results from toxin production (22). In order to analyze the complex virulence signal pathway in V. dahliae, the toxic protein extracted from V. dahliae has been studied. This toxin can cause the rupture of the cell membrane of the host plant, change the osmotic pressure, lead to cell death, and cause plant disease (23). For example, V. dahliae toxin reduced the growth of cotton callus (24). Moreover, defense gene *GhMLP* can be induced by V. dahliae toxin in root of cotton plant (25).

Hydrophobic protein located on the cell wall of fungi is one of the guaranty for fungal survival, which is a class of small molecule proteins rich in cysteine (26). In the *THN* defect of airborne mycelium *Schizophyllum*, a class of small proteins related to cell wall hydrophobicity is called hydrophobic proteins. Most of them contain eight conserved cysteine residues, which pair to form four disulfide bonds. Cysteine residues are important for the solubility of hydrophobic protein SC3 in *Schizophylla* (27). It was reported that *MPG1*, as a hydrophobin gene, affected the virulence, conidiation, and appressorium formation in Magnaporthe grisea (28). In addition, *HFB4* and *HFB10* could affect the spore-mediated diffusion process; they encapsulated spores and mediated environmental interactions (29). Two hydrophobic genes (*Pgh1 and Pgh2*) were associated with the growth and development of *Phlebiopsis gigantea* (30). In V. dahliae, deletion of hydrophobin gene *VDH1* could reduce many physiological activities of micronucleus development and spore viability (8, 31). Recently, the gene expression was studied during the development and pathogenic growth of the sclerotium in V. dahliae. The expressed sequence tags (EST) analysis had been done during the microsclerotia development and pathogen growth, including hydrophobin genes (32). In the process of interaction between pathogen and plant, hydrophobic protein can promote plant growth and improve plant disease resistance by inducing plant growth and the expression of disease resistance genes (28, 30).

In the present study, a hydrophobic protein VdHP1 with 47% hydrophobic amino acid was identified in V. dahliae, which contained the transmembrane domain and the signal peptide. The deletion mutants were highly hydrophobic, but complementary mutants were hydrophilic. In addition, the deletion mutants conferred higher resistance to Congo red (CR), UV, and high temperature, and hypersensitivity to NaCl. Different carbon source assay showed that the utilization ability of skim milk, cellulose, and starch was greatly enhanced in *ΔVdHP1* strains. Knocking down of *VdHP1* did not affect mycelium penetration on cellophane but contributed to mycelium growth on surface of the living plant cells, leading to a significant increase in pathogenicity of V. dahliae in cotton. These data highlighted that the hydrophobins gene *VdHP1* negatively regulated the virulence of V. dahliae, and played an important role in development, adaptability, and pathogenicity in V. dahliae.

## RESULTS

### VdHP1 cloning and sequence analysis.

**FIG 1:**
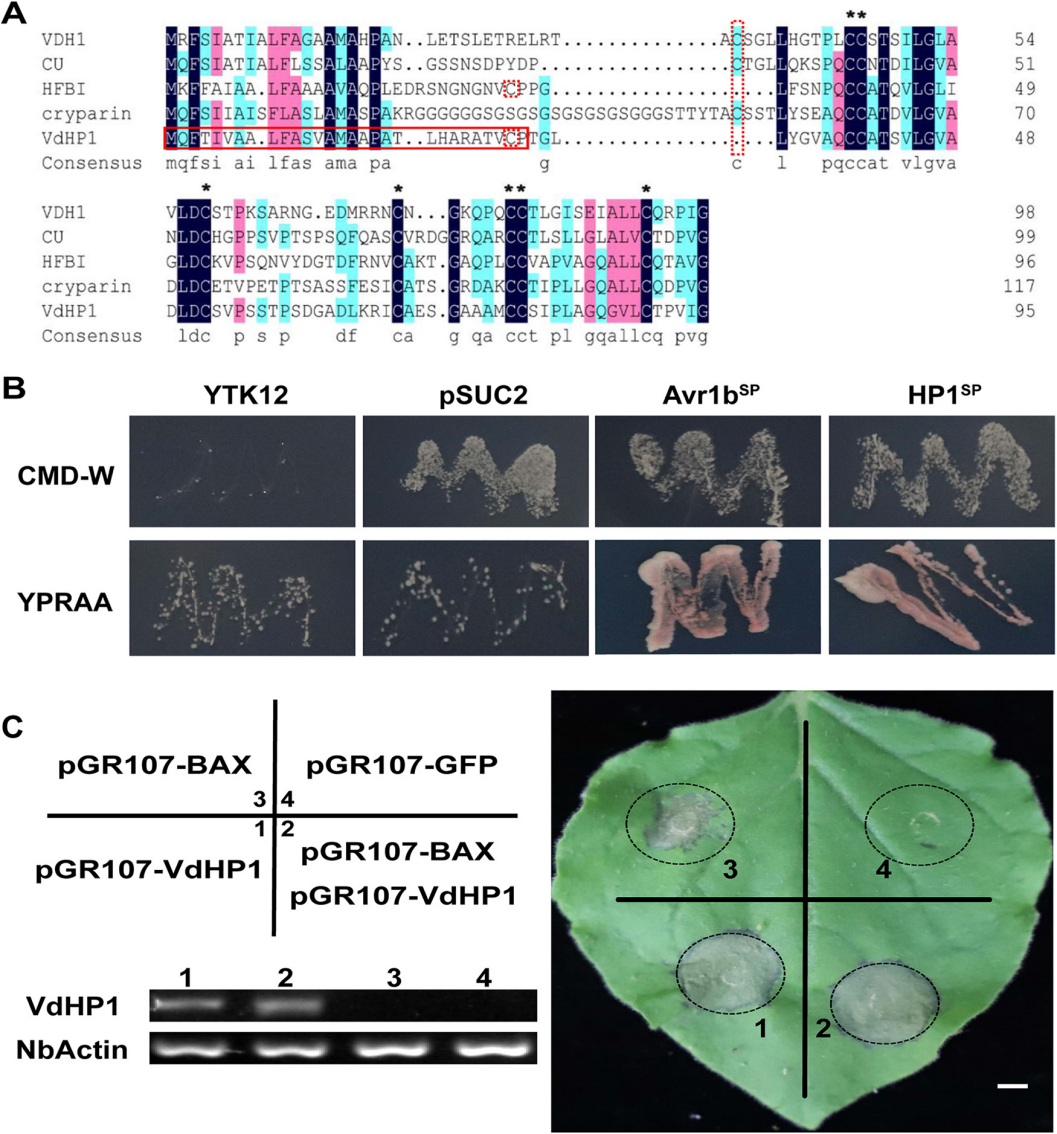
VdHP1 was a hydrophobic protein and induced hypersensitive response. (A) The VdHP1 amino acid sequence aligned between VdHP1, cerato-ulmin from *O. ulmi* (80), *T. reesei* HFB1 (81), cryopyrin from *C. parasitica* (82), and *VDH1* from V. dahliae (8, 31). Identical residues were shaded black, similar residues were shaded blue or pink, and cysteine residues were marked with asterisks or red dotted box. The signal peptide of VdHP1 was marked by a solid red box. (B) Conformation of the function of the signal peptide of VdHP1 by yeast secretion trap assay. (C) VdHP1 induced hypersensitive response on N. benthamiana leaves. The numbers 1, 2, 3, and 4 represent a one-to-one correspondence in gels. Semi-quantitative RT-PCR (sqRT-PCR) analysis of transiently expressed *VdHP1* in N. benthamiana leaves. *NbActin* was used as the control. The gels are agarose gels (0.1%). Scale bar = 0.5 cm.

A hydrophobic protein (VDAG_08956) was identified in V. dahliae strain VdLs.17 (https://www.ncbi.nlm.nih.gov/genome/832), which has a typical Hydrophobin_2 domain (pfam06766) and was tentatively named VdHP1. We cloned the full-length cDNA of *VdHP1* from the strong pathogenic defoliating strain Vd080, which contained 288 bp, and encoded a protein of 95 amino acids with an N-terminal signal peptide sequence, a transmembrane domain, and a C-terminal Hydrophobin_2 domain (Fig. S1). Phylogenetic analysis performed with the protein sequences showed that the VdHP1 was divided into class II (Fig. S2). It had low similarity comparison of the VdHP1 amino acid sequence with those of known fungal hydrophobins. But the hydrophobic amino acid content of VdHP1 was as high as 47%, while *VDH1* only has 37% hydrophobic amino acid with eight conservative cysteines (Fig. 1A); these showed that the fungal hydrophobic proteins have low homology but high hydrophobic amino acid content.

The yeast signal trap system assay showed that YTK12-pSUC2-VdHP1^SP^ had been ensured the normal growth on CMD-W and YPRAA medium, which suggested that VdHP1 was most likely secreted into the extracellular space during infection (Fig. 1B). Then, cell death experiments were performed in Nicotiana benthamiana leaves. Plant hypersensitive response (HR) was induced by separate injections of VdHP1 and BAX, respectively, but HR had not been suppressed by co-expressing BAX and VdHP1 (Fig. 1C). These results showed that VdHP1 could cause cell death in plants, suggesting that VdHP1functions as an effector to induced plant immunity.

### Generation of VdHP1 mutants.

**FIG 2:**
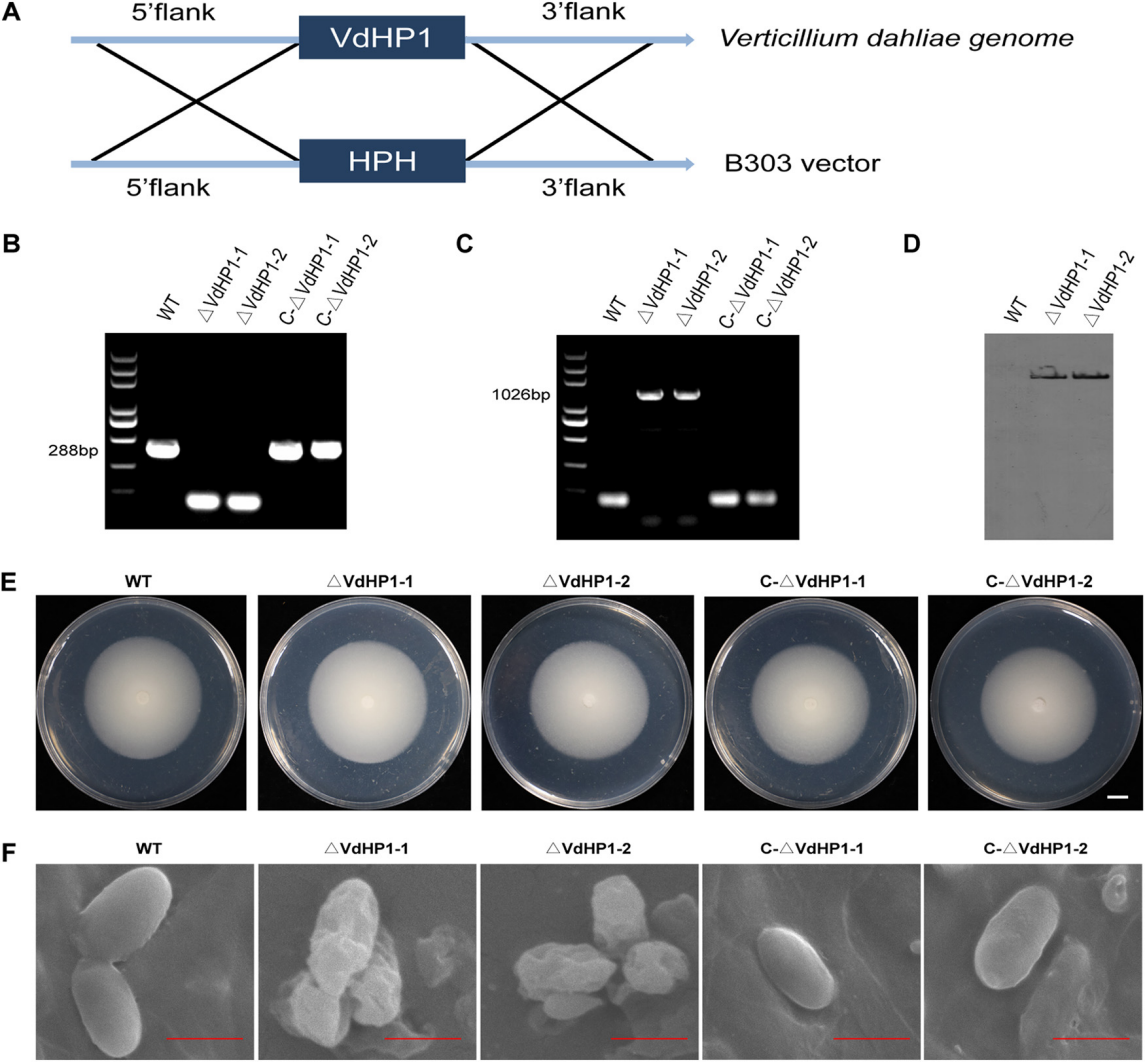
Strategy for construction of gene deletion vector and identification of *VdHP1* mutants. (A) The strategy for homologous recombination of *VdHP1* in V. dahliae. (B) and (C) The confirmation of deletion and complemented strains by PCR. (D) Southern hybridization analysis. Probe: Hyg; DNA of WT, *ΔVdHP1*-*1*, and *ΔVdHP1-2* were digested by HindIII. (E) Phenotype of all mutants on PDA at 10 dpi. Bar = 1cm. (F) The conidia of the morphology of WT, *ΔVdHP1*, and *C-ΔVdHP1* strains under scanning electron micrographs. Scale bar = 4 μm.

To investigate the function of *VdHP1* in the development and pathogenicity of V. dahliae, *VdHP1* was knocked out in V. dahliae Vd080 genome through the homologous recombination method (Fig. 2A). Two independent deletion mutants (*ΔVdHP1-1*, *ΔVdHP1-2*) were obtained and verified by PCR (Fig. 2B). Southern hybridization showed that the Hyg was a single copy in two deletion mutants (Fig. 2D). Furthermore, complementation mutants were obtained in the background of knockout mutants by reintroduction with the wild-type *VdHP1* copy. Both complementary mutants (*C-ΔVdHP1-1*, *C-ΔVdHP1-2*) were confirmed by PCR with the primer pair Hyg-F/Hyg-R (Fig. 2C). Compared with wild type (WT), the deletion and complementary mutants were not phenotypically different after growth on PDA (Fig. 2E). However, the conidia of two deletion mutants were deformed, with shrinkage surfaces. While the conidia of WT and the complementary mutants were oval and the surface was smooth (Fig. 2F). All strains were grown on cellulose membrane in PDA medium for 6 days. After opening the cellulose membrane and growing for 6 h postinoculation (hpi), the microsclerotia of deletion mutants were less than that of WT and the complemented strains (Fig. S3).

### VdHP1 affects the hydrophobic function of V. dahliae.

**FIG 3:**
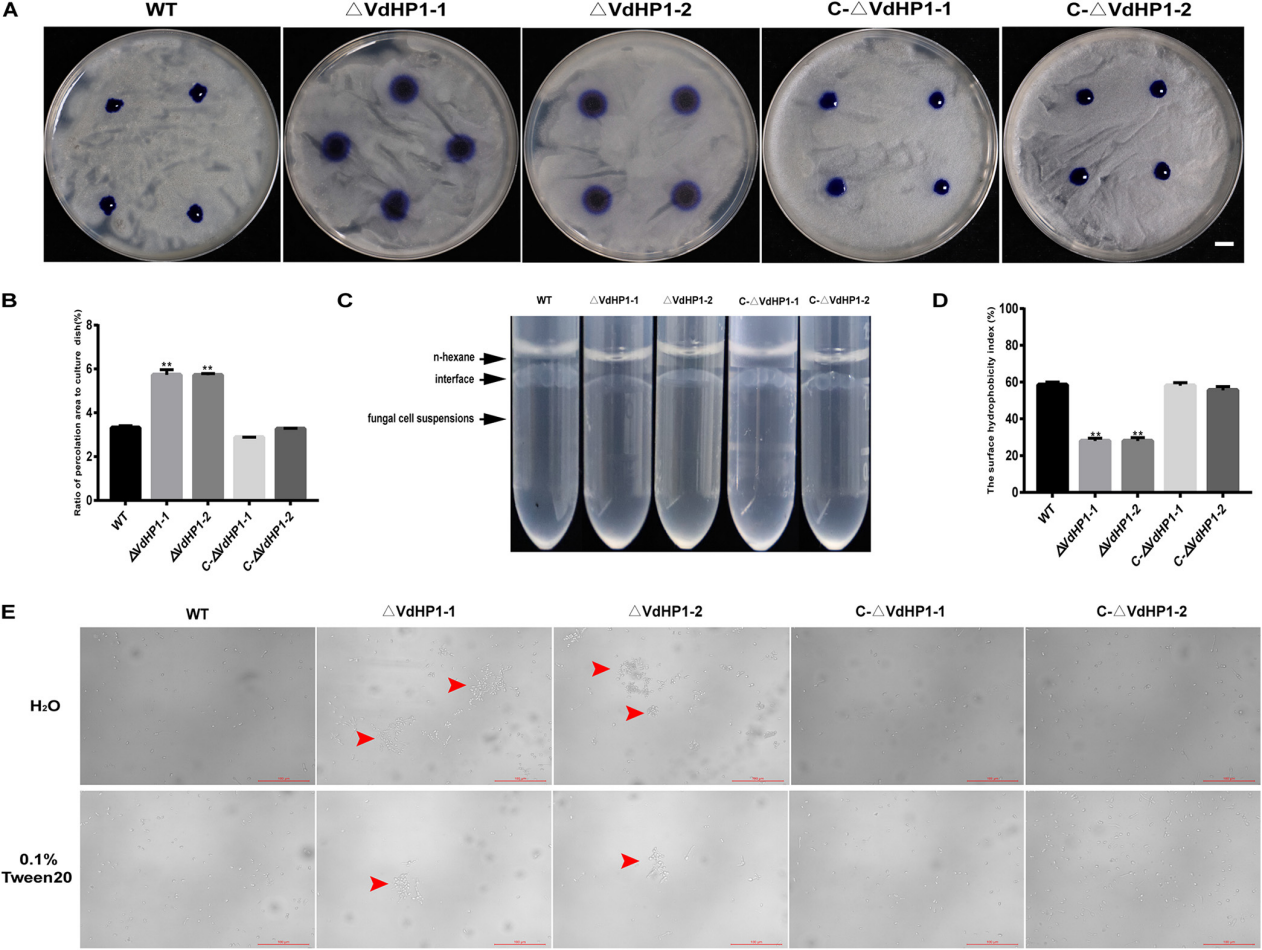
Comparison of hydrophobicity between WT and mutants. (A) The penetration of various strains to aqueous aniline blue. Bar = 1 cm. (B) Percentage of infiltration area to total petri dish area. Quantitative calculation was performed by Image-Pro Plus (Media Cybernetics, Rockville, MD, USA) software. (C) Surface-displaying hydrophobicity of WT and mutants by the state of the interface between organic phase and water phase. (D) The surface hydrophobicity index of WT, *ΔVdHP1*, and *C-ΔVdHP1* strains conidia. (E) Images of conidial suspensions of WT and mutants suspended in H_2_O and 0.1% Tween 20 under optical microscope. Red arrows show conidia clustering.

To investigate the function of VdHP1 in the hydrophobicity of V. dahliae, 20 μL of 0.5% aqueous aniline blue was dropped on the hyphal surface of colonies. The results showed that *ΔVdHP1-1* and *ΔVdHP1-2* were easily penetrated by the aqueous Aniline Blue than that of WT, *C-ΔVdHP1-1*, and *C-ΔVdHP1-2* (Fig. 3A). The penetrated area of deletion mutants was nearly doubled compared with WT and complemented strains (Fig. 3B). In addition, the interface between *ΔVdHP1* strains suspension and n-hexane recovered to a clear and smooth boundary, while the interface between WT, *C-ΔVdHP1* strains suspension and n-hexane still maintained an obvious “oil-drop” layer (Fig. 3C). The cell surface hydrophobicity of all strains was assessed by MATH assay. It was shown that the surface hydrophobicity of *ΔVdHP1* strains was ∼ 29%, much lower than that of WT and complemented strains (Fig. 3D). The spores of *ΔVdHP1* strains clustered together in large numbers to form a “clump-like” structure, which did not disappear even in the presence of surfactants (0.1% Tween 20). Meanwhile, the spores of WT and complemented strains had been scattered open (Fig. 3E). These results showed that the deletion of *VdHP1* gene resulted in decreased the hydrophobicity of V. dahliae, suggesting *VdHP1* positively affects the hydrophobicity of V. dahliae, and played an important role in the dispersion of conidia mediated by liquid.

### The *VdHP1* mutation more is resistant to stress.

**FIG 4:**
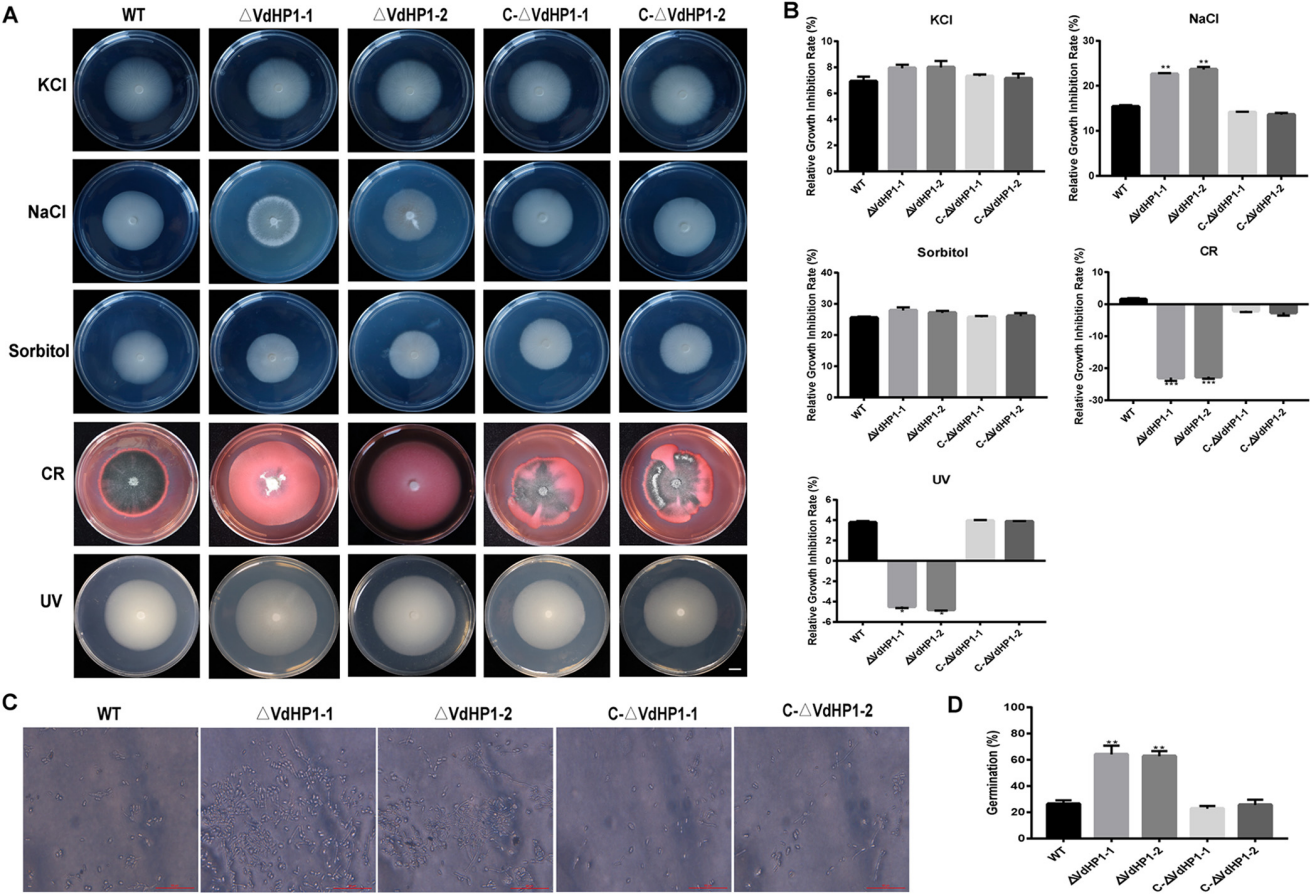
*VdHP1* plays an important role in osmotic stress resistance in V. dahliae. (A) Phenotypes of the WT, *ΔVdHP1*, and *C-ΔVdHP1* strains grown on PDA supplemented with 0.4 M NaCl, 0.8 M KCl, 1.2 M sorbitol, 50 mg/mL Congo red (CR) and treated by UV 10 s for 15 days. Scale bar = 1 cm. (B) Bar charts show relative growth inhibition rate of strains in (A). (C) The conidia of WT and mutant strains conidia after heat shock at 45°C for 1 h followed by plating on PDA after 22 h at 25°C. (D) The conidia germination percent of WT and mutants in (C). Error bars represent the standard deviation of three replicates, determined in multivariate analysis of variance comparison with the WT. Values represent means standard deviation of three replicates. *, *P* < 0.05; **, *P* < 0.01; ***, *P* < 0.001.

Compared with the WT, *ΔVdHP1* strains exhibited no obvious growth defect on PDA (Fig. 2E). The colony diameter of *ΔVdHP1* strains were obviously reduced compared with that of WT and complemented strains in the presence of NaCl (Fig. 4A). The relative growth inhibition rate of *ΔVdHP1-1* and *ΔVdHP1-2* was 22.59% and 23.63%, respectively (Fig. 4B). However, in the presence of KCl and sorbitol, *ΔVdHP1* was not significantly different in relative growth inhibition rate (Fig. 4A and B). We deduced that the *ΔVdHP1-1* and *ΔVdHP1-2* strains did not exhibit hypersensitivity to KCl and sorbitol. Interestingly, the deletion strains showed in significantly enhanced resistance to CR and UV treatment (Fig. 4A and B). When the conidia of WT or *C-ΔVdHP1* strains were heat shocked for 1 h at 45°C, only ∼ 25% of the conidia germinated on PDA at 25°C for 22 h. In contrast, 62% ∼ 64% *ΔVdHP1-1* and *ΔVdHP1-2* conidia germinated, indicating that *ΔVdHP1* strains were more thermotolerant than the WT and *C-ΔVdHP1* strains (Fig. 4C and D). Taken together, the deletion of *VdHP1* strains caused hypersensitivity to NaCl, but which was relative insensitivity to KCl and sorbitol, resistance to CR, UV, and high temperature, which suggested that *ΔVdHP1* mutation were more resistant to stress.

### Determination of efficiency of utilizing different carbon source of *ΔVdHP1* strains.

**FIG 5:**
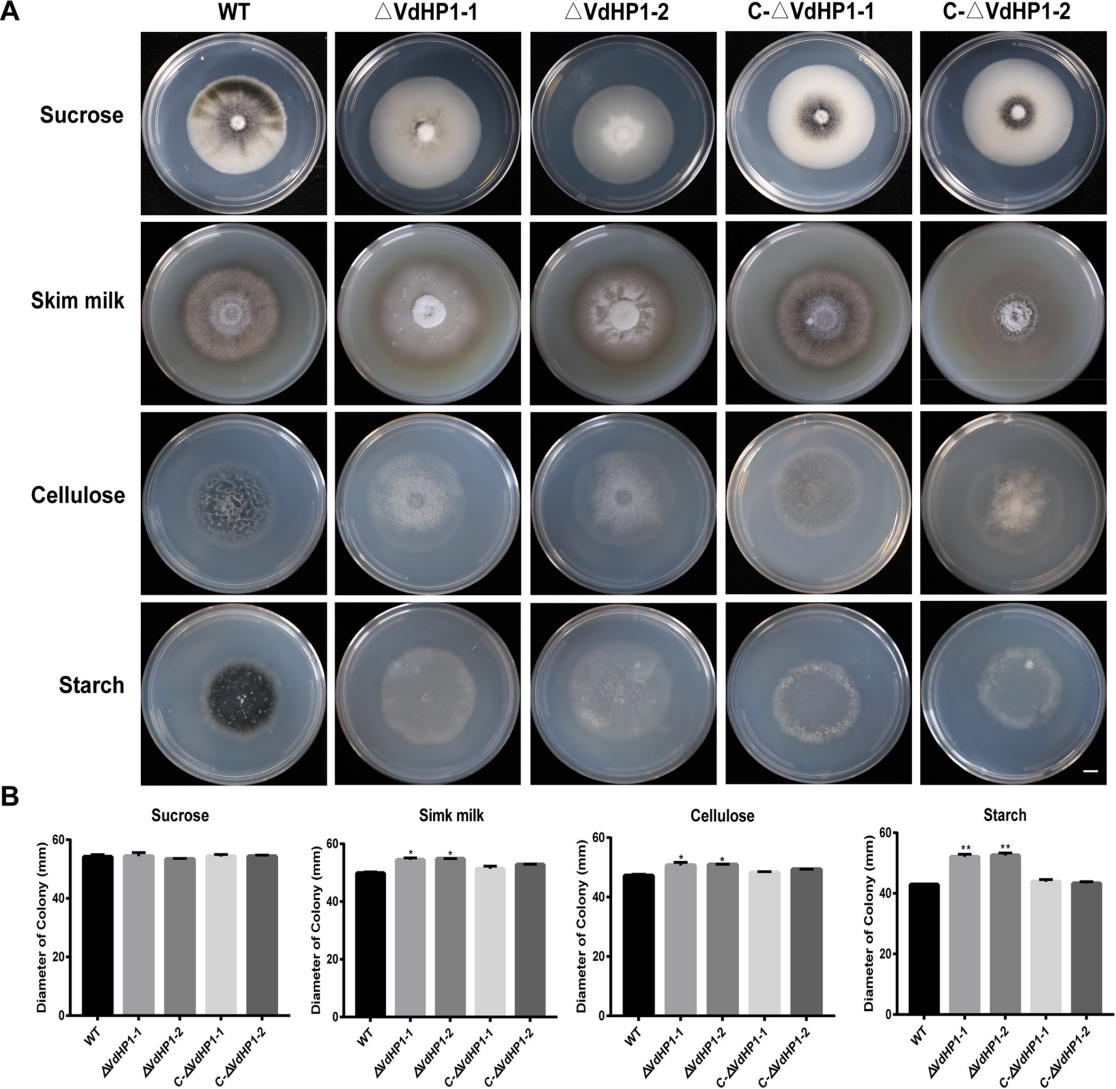
*VdHP1* plays an essential role in the utilization of different carbon sources. (A) Culture characteristics and growth rates of isolates on media with different carbon sources. Scale bar = 1 cm. (B) Radial growth of all strains. The diameter data was obtained by measuring the diameter of each colony grown on the medium. Error bars represent the standard deviation of three biological replicates. *, *P* < 0.05; **, *P* < 0.01.

The colonization and invasion of V. dahliae on plant rhizomes were closely related to its ability to decompose and utilize different carbon sources. The mycelial growth of WT and mutant strains was measured on Czapek solid medium with sucrose, skim milk, cellulose, and starch as the sole carbon source, respectively. The results showed that the colony diameter of all strains were not significantly different on medium containing sucrose (Fig. 5A). But, the colony diameter of *ΔVdHP1* strains was significantly increased on medium with starch, cellulose, and skim milk (Fig. 5A). Especially, in the presence of starch, the relative growth of *ΔVdHP1* strains was increased with ∼ 22.57% (Fig. 5B). The results implied *VdHP1* might negatively regulate the decomposition and utilization of skim milk, cellulose, and starch in V. dahliae.

### *VdHP1* did not affect mycelium penetration but contributed to mycelium growth.

**FIG 6:**
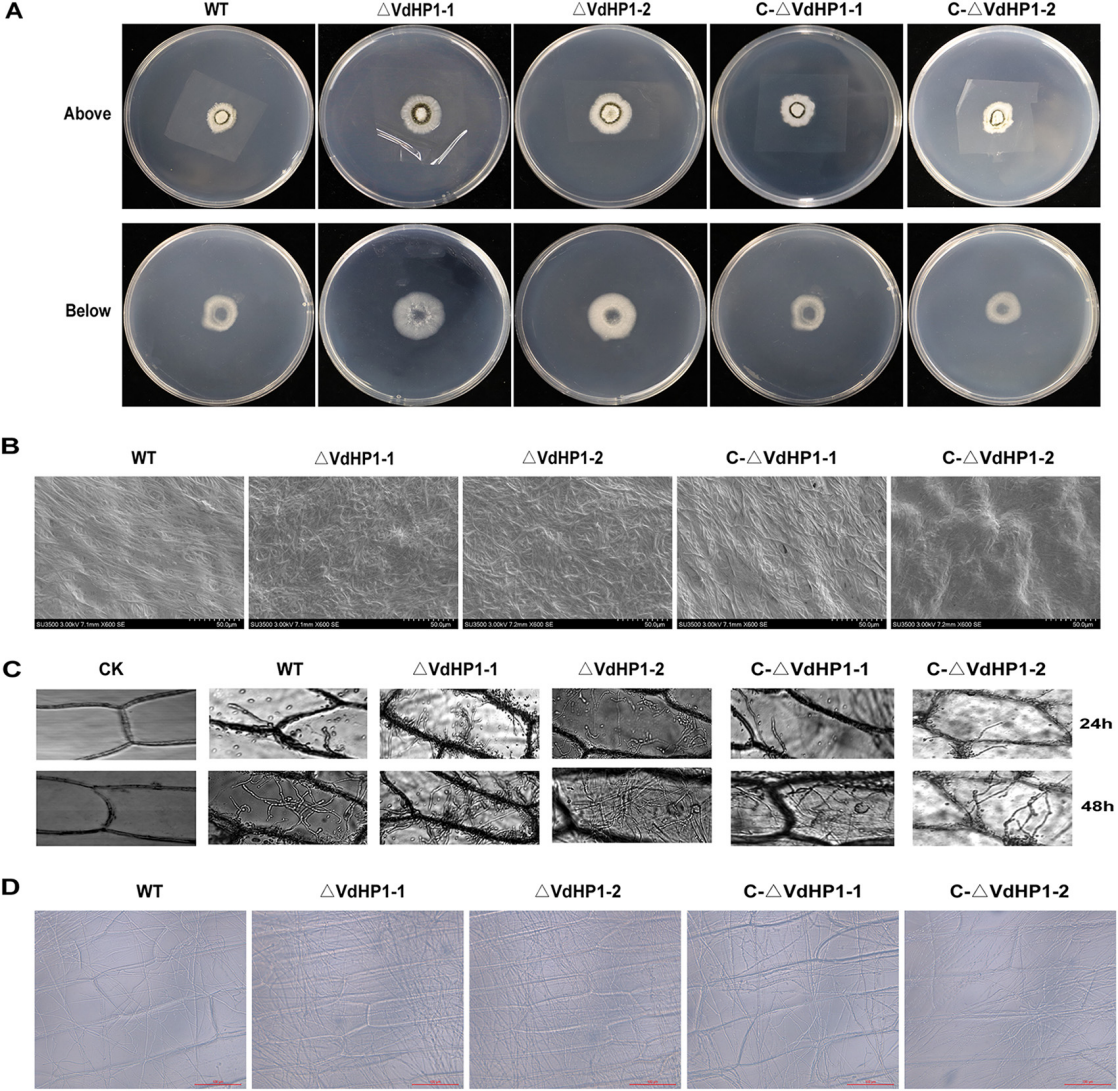
The cellophane and onion epidermis penetration assay. (A) All the strains were grown on cellophane for 3 dpi (above) and removal of the cellophane for 3 dpi (below). Scale bar = 1 cm. (B) Observation of the mycelium development on cellophane at 3 dpi. Scale bar = 50 μm. (C) Infection assays of onion epidermis examined at 24 h and 48 h. (D) Mycelium development on onion epidermis examined at 3 dpi. Error bars represent standard errors.

To observe the ability of all strains to penetrate cellophane, equal amounts of conidia of each strain were cultured onto the cellophane overlaid on PDA medium for 3 days. The results showed that the deletion mutants had similar colony morphology compared with WT and complemented strains. The colony diameter of WT and the mutants grew normally on the cellophane (Fig. 6A). After the cellophane was removed and further cultured for 3 days postinoculation (dpi), there were obvious differences between the deletion mutants and WT strains. The hyphae of deletion mutants had proliferated faster than that of WT and complemented mutants (Fig. 6A). The hyphae on the cellophane was observed under a scanning electron microscopy. The mycelium of WT and complemented strains showed normal and uniform, while the mycelium of deletion strains displayed dense and crowded (Fig. 6B). In addition, the mycelia of *ΔVdHP1* strains grew quickly and densely on epidermal onion compared with the WT and complemented strains (Fig. 6C and D). These results indicated that the *VdHP1* did not affect mycelium penetration but also contributed to mycelium growth of V. dahliae.

### *VdHP1* negatively regulates virulence of V. dahliae.

**FIG 7:**
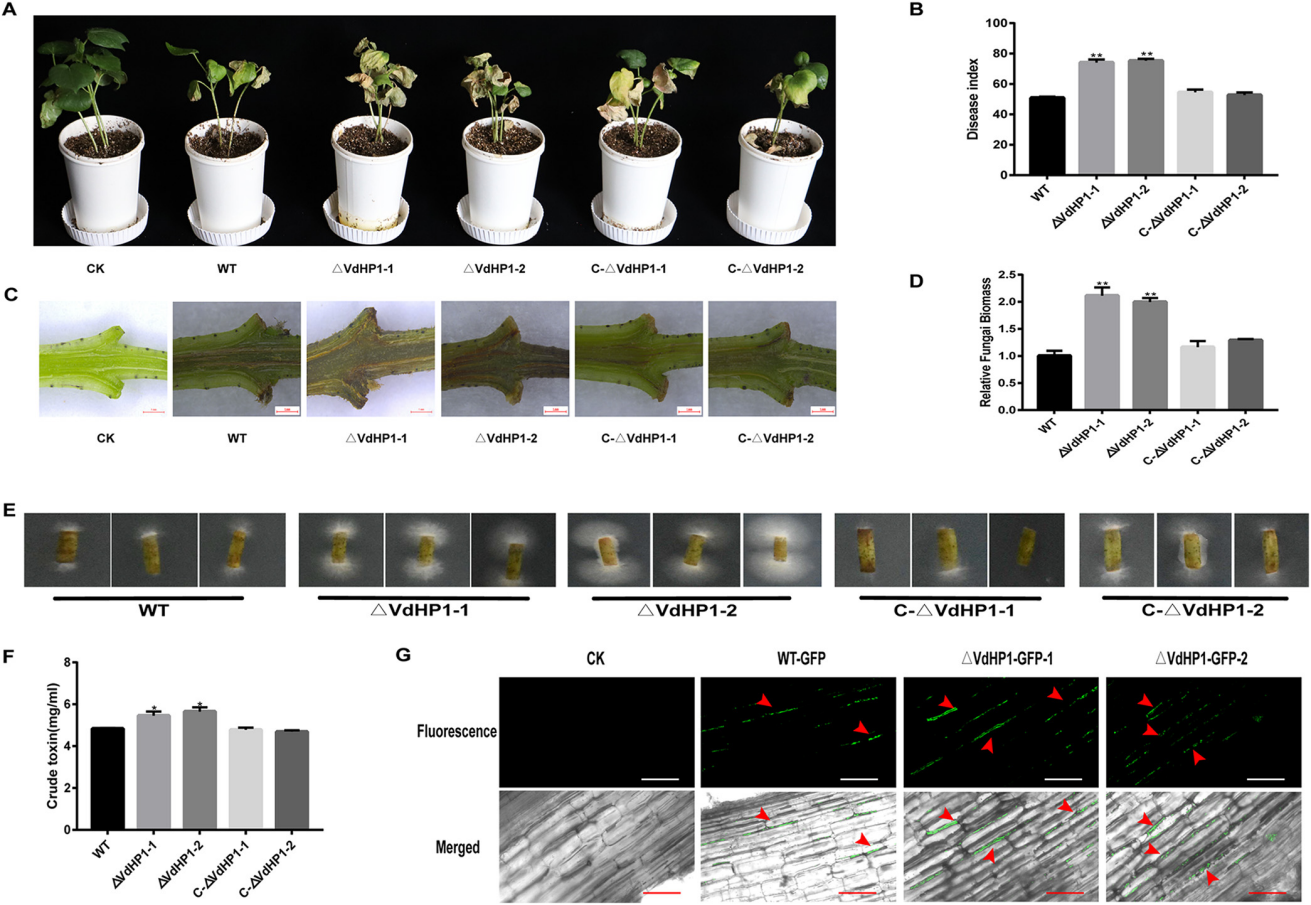
*VdHP1* negatively regulated the pathogenicity of V. dahliae. (A) Disease symptoms of cotton plants at 25 dpi after WT, *ΔVdHP1*, and *C-ΔVdHP1* strains infection. (B) The DI of cotton plants at 25 dpi after WT, *ΔVdHP1*, and *C-ΔVdHP1* strains infection. (C) Vascular discoloration of stems tissues of cotton seedlings. (D) Quantification of fungus DNA in cotton stems at 21 dpi. *Vdβt* was used as the detection gene, and the upland cotton *Act* gene was used as the endogenous control gene. (E) Reisolation of V. dahliae strains from the stem of cotton plants at 25 dpi for 5 days on PDA medium. (F) The crude toxin content of WT, *ΔVdHP1*, and *C-ΔVdHP1* strains. (G) The colonization of WT-GFP, *ΔVdHP1-GFP-1*, and *ΔVdHP1-GFP-2* in cotton root. Scale bar = 50 μm.

To examine the role of VdHP1 in virulence, the pathogenicity test was performed in the susceptible *G. hirsutum* “Jimian11” by infection with the WT, *ΔVdHP1* strains, and *C-ΔVdHP1* strains. The results showed that the cotton seedings were seriously wilting, yellowing, and even had fallen leaves after inoculation by the conidial suspensions of *ΔVdHP1* strains; while the cotton plants infected by WT and *C-ΔVdHP1* strains showed slight wilting symptoms (Fig. 7A). At 25 days after inoculation, the disease index (DI) of cotton plants infected by *ΔVdHP1-1* and *ΔVdHP1-2* strains were about 74.16 and 75.31, respectively, while that of cotton plants infected by WT, C-*ΔVdHP1-1* and C-*ΔVdHP1-2* were only about 51.00, 54.55, and 52.67, respectively (Fig. 7B). Compared with the cotton plants infected with WT and *C-ΔVdHP1* strains, the vascular bundles of cotton plants inoculated with *ΔVdHP1* strains had more severe browning (Fig. 7C). The fungi biomass in the stem tissue of cotton plants inoculated with *ΔVdHP1* strains was higher than that of cotton plants infected with WT and complemented strains (Fig. 7D). As expected, fungal hyphae were recovered from plants inoculated with V. dahliae, the deletion strains were reisolated from vascular tissue at a higher rate of isolation than those of WT and the complemented strains (Fig. 7E). The crude toxin content of *ΔVdHP1* strains was significantly increased compared with the WT and complementary strains (Fig. 7F). In addition, the colonization and dispersal of *ΔVdHP1* strains in cotton roots were also increased compared with the WT (Fig. 7G). These results showed that *VdHP1* negatively regulated the virulence of V. dahliae.

## DISCUSSION

Fungal hydrophobic proteins are a type of small-secreted amphiphilic proteins produced by filamentous fungi, which play an important role in the fungi growth and development (33). These proteins are divided into two types, class I and class II, according to the hydropathy patterns and stability of films formed by hydrophobins. Class II hydrophobins films can be dissolved in detergents like Tween 20 (34). Compared with the films formed by class II hydrophobins, class I hydrophobins form amyloid-like rodlets that are highly insoluble in water, organic solvents, and detergents (35). Furthermore, hydrophobins have low homology but high hydrophobic amino acid content (36). The hydrophobin HGFI (derived from *Grifola frondosa*) and HFBI (derived from Trichoderma reesei) have only 18% homology, but their hydrophobic amino acid content exceeds 30% (37–40). In this study, VdHP1 was identified as a hydrophobin from V. dahliae, which was clustered into the class II hydrophobins. The VdHP1 has low homology with other class II hydrophobins but has 47% hydrophobic amino acid.

During colonization of their hosts, pathogens secreted effector proteins to promote disease development through various mechanisms, and also regulate the plant immune response to pathogens (41). The V. dahliae fungus releases or secretes nearly 800 proteins (42). Effector Vd424Y triggered cell death and was necessary for pathogenicity in *V. dahiae* (43). Among other fungi, Aspergillus oryzae could tolerate ion stress, maintain cell wall integrity and virulence due to the regulation of A. oryzae *CRZ1* gene (44). HYTLO1, secreted by *Trichoderma longibrachiatum*, could trigger calcium signaling pathway to enhance *Lotus japonicus* resistance (45). In addition, both types of hydrophobic proteins had been reported to play a role in the interaction of pathogens and host or environment (46). The hydrophobin-Like OmSSP1 within Class I hydrophobins reduced capacity to form ericoid mycorrhiza with *Vaccinium myrtillus* roots (47). From *T. asperellum*, the class II hydrophobin gene *HFBII-4* enhanced poplar resistance to *A. alternate* (48). Due to VdHP1 having secretory properties (Fig. S2B and C), transient expression in N. benthamiana leaves was carried out and could induce the hypersensitive response, but VdHP1 was not suppressed by the BAX-mediated cell death (Fig. 1C). It was revealed that VdHP1 had induced plant HR, suggesting that VdHP1 function as effector to activate plant immunity.

Fungal hydrophobic proteins were involved in many biological processes, such as the formation of fungal cell wall, airborne mycelia, and spores (49). Hydrophobicity of hydrophobic protein could help the fungi attach to the host surfaces (39, 50). But, the result on hydrophobicity of a hydrophobin deletion on different fungi could vary in influence and importance. Loss of *RodA* displayed altered electrostatic characteristics, with slight alterations on cell hydrophobicity in Aspergillus Fumigatus spore (51). Loss of *Hyd1* or *Hyd3* did not influence mycelial hydrophobicity in *Clonostachys rosea*. However the conidia from the double deletion mutant *ΔHyd1 and ΔHyd3* clumped together in solution and had lower hydrophobicity index than the WT (34). For V. dahliae, our data indicated that loss of VdHP1 resulted in increased hydrophilicity, and conidia clumped together and could not disperse effectively (Fig. 3C and E). Overall, *VdHP1* affected the hydrophobic function of V. dahliae.

Microsclerotia, hypha, and spores all played important roles in the life cycle of V. dahliae (52). Loss of *VdCmr1* eliminated melanin biosynthesis was detectable but did not affect microsclerotia development. Deletion of *MPG1* severely influenced total virulence because of an inability to form appressoria in *M. grisea* (28). *VDH1* is involved in protection against environmental stress, and played a role in morphogenesiss, but no role in the virulence of V. dahliae (8, 40). Here, deletion of *VdHP1* strains exhibited inhibited microsclerotial formation and reduced spores smoothness. Although *ΔVdHP1* strains showed similar colony morphology compared with WT and complemented strains on PDA, the mycelia of *ΔVdHP1* strains grew quickly and more densely after penetration of cellophane or on epidermal onion. This indicated that the *VdHP1* did not affect mycelium penetration but also contributed to mycelium growth in the process of initial colonization of V. dahliae.

Previously, studies characterized *VdHog1*, *VdPbs2*, *VdSsk1*, and *Vdssk2* of V. dahliae, as playing an important role in resistant to osmotic agents and CR (18, 52, 53). In the presence of CR, high expression of genes associated with cell wall biosynthesis was caused by deleting *VdSsk1* or *VdSsk2* (18, 52). Moreover, cryopyrin owns the lectin-like properties, and binding to the cell wall occurred in submerged culture in the filamentous ascomycete *Cryphonectria parasitica* (54). Overcoming the natural barrier of the cell wall was a key step for fungal pathogens to infect plant hosts. Hydrolytic cell wall-degrading enzymes played an important role in the pathogenicity of pathogens by degrading the plant cell wall. F. oxysporum and *C. carbonum* could degrade plant cell polysaccharides by producing proteases, pectinases, and cellulases (55, 56). Proteases, pectinases, and cellulases allowed pathogens to use plant proteins as a source of nutrients (57). In the study, the *ΔVdHP1* strains showed normal sensitivity to NaCl, but had relative insensitivity to KCl and sorbitol, and was resistance to CR, UV, and high temperature, which suggested that *ΔVdHP1* was more resistant to stress (Fig. 4). In addition, after the knockout of *VdHP1* gene, the utilization ability of skim milk, cellulose, and starch was improved in V. dahliae. In brief, the *ΔVdHP1* may be more adaptable and able to survive in the natural environment.

Hydrophobic proteins were also involved in the regulation of pathogenicity. Previous studies have shown that fungal hydrophobin played distinct functions in the interaction between pathogens and hosts, and were an important group of proteins from a biological standpoint. Filamentous fungi could affect their environment to promote growth by the means of hydrophobic proteins (58). Deletion of *Fghyd2* and *Fghyd3* in F. graminearum, reduced the symptomatic spikelets in wheat (59). *VdSkn7*-deficient mutants displayed severe growth defect under heat shock, cell wall perturbing agents and H_2_O_2_, and were significantly less virulent but were not sensitive to osmotic stresses compared with the WT (60). The pathogenicity of *ΔCgHYD1*, a hydrophobin gene, was significantly stronger in rubber tree than that of WT (61). In this study, it was found that the cotton seedings after inoculation by the conidial suspensions of *ΔVdHP1* strains showed severe wilting and chlorosis of the leaves and even defoliation. The crude toxin content of *VdHP1* deletion mutants was significantly increased compared with the WT and complementary strains. The colonization and dispersal of *ΔVdHP1* strains in cotton roots were also increased. It was inferred that *VdHP1* was a negative regulator of virulence, and correlated with pathogenicity of V. dahliae.

In summary, our results suggested that hydrophobin gene *VdHP1* of V. dahliae could induce cell death and activate plant immune responses. VdHP1 affected the hydrophobicity of V. dahliae. Loss of VdHP1 gene resulted in increased hydrophilicity, inhibited microsclerotial formation, and reduced spores smoothness. VdHP1 may be also involved in regulating the decomposition and utilization of skim milk, cellulose, and starch during mycelial growth. *ΔVdHP1* has stronger resistance to abiotic stress. *VdHP1* did not affect mycelium penetration on cellophane but contributed to mycelium growth on surface of the living plant cells. *VdHP1* negatively regulated the total virulence of V. dahliae, and enhanced the pathogenicity of pathogens. This study suggests that the hydrophobic proteins were importance in development, adaptability, and pathogenicity of V. dahliae, which may provide a new viewpoint for us to further understand the molecular mechanisms of pathogen virulence.

## MATERIALS AND METHODS

### Fungal strain, plant material, and culture conditions.

The wild type V. dahliae strain Vd080 is a strong pathogenic defoliating strain. It was isolated and purified from cotton collected in Xinji, Hebei, China, and was provided by the Institute of Cotton Research of Chinese Academy of Agricultural Sciences. The fungus was cultured in liquid Czapek Dox medium or on potato dextrose agar medium (PDA) (62).

Upland cotton (*Gossypium hirsutum*) cultivar “Jimian11” was highly susceptive to V. dahliae (63), and was grown in a greenhouse under 8 h/16 h dark/light cycle at 26°C with a relative humidity of 60%. N. benthamiana was grown in a greenhouse under a 8 h/16 h dark/light cycle at 22 ± 1°C.

### Gene cloning and bioinformatics analysis.

The full length of coding sequences of *VdHP1* (VDAG_08956) was amplified from cDNA of V. dahliae Vd080 using the specific primers (Table S1). Multiple sequence alignments were performed using DNAMAN. CDD tool (https://www.ncbi.nlm.nih.gov/Structure/cdd/wrpsb.cgi) was used to predict the functional conserved domain of VdHP1. The secretory characteristics of the protein encoded by *VdHP1* gene was predicted by online software (http://www.cbs.dtu.dk/services/TMHMM/). A potential signal peptide was predicted by the signal peptide prediction server SignalP5.0 (http://www.cbs.dtu.dk/services/SignalP/index.php). The phylogenetic analysis of VdHP1 and other hydrophobins was performed by the MEGA7 software using neighbor-joining method (64).

### Yeast signal sequence trap system and cell death assay.

Yeast signal sequence trap system was performed to verify the function of predicted signal peptide of VdHP1 (65). The predicted signal peptide of VdHP1 was inserted into the vector pSUC2. The signal peptide of Avr1b was inserted into the pSUC2, as the positive. Both pSUC2-HP1^SP^ and pSUC2-Avr1b^SP^ were transformed into the yeast strain YTK12. Then, they were screened on CMD-W medium. The positive transformants were incubated on YPRAA medium (2% raffinose). The empty pSUC2 vector was used as the negative control. The primers used were listed in Table S1.

The full length of coding sequences of *VdHP1* (with the signal peptide sequence) was inserted into pGR107-GFP vector and transformed into A. tumefaciens GV3101 by heat shock. The transformants were identified by PCR. The suspension of pGR107-GFP and pGR107-BAX were negative and positive controls, respectively (66). And pGR107-GFP, pGR107-BAX and pGR107-*VdHP1* were transient expressed on N. benthamiana leaves by injection, respectively. Meanwhile, pGR107-BAX and pGR107-*VdHP1* were co-expressed on N. benthamiana leaves by co-injection with 1:1. The N. benthamiana leaves were collected and observed. Each assay was performed on three leaves from three individual plants, and repeated at three times.

### Target gene knockout and complementation.

*VdHP1* deletion mutants and complementary mutants were obtained by protoplast transformation (67, 68). The upstream Up-1.1Kbp and downstream Down-1.1Kbp sequences of *VdHP1* gene were selected. PCR was amplified in WT genomic DNA with the following primer pairs: B303-VdHP1-UP-F/R, B303-VdHP1-DOWN-F/R (Table S1). The hygromycin resistant fragment (Hyg) in vector B303 was cloned with Hyg-F/R (Table S1). These fragments were fused with linearized B303 vector using recombinase (ClonExpress Ultra One Step Cloning Kit, Vazyme, Nanjing, China) to construct Up-Hyg-Down-B303-Hyg transformation plasmid according to the manufacturer’s instructions. The transformation of bacterial plasmid DNA was carried out by the standard chemical scheme.

Up-Hyg-Down-B303-Hyg was transformed into protoplasts of V. dahliae. The positive colonies (*ΔVdHP1*) were screened on PDA containing hygromycin, and confirmed by PCR with the primers Hyg-F/R. The copy of hyg was confirmed by Southern Blotting. Fragments of Up, *VdHP1*, Down, and linearized pCAM-BIA1302-neo plasmid was cloned together using the same way to obtain the Up-VdHP1-Down- pCAMBIA1302 fusion plasmid. Then, it was transformed into the protoplast of the knockout mutant strains for subsequent selection. Transformants were selected based on the vector antibiotic resistance, and confirmed by PCR with the primers *VdHP1*-F/R.

To construct the *ΔVdHP1-GFP* strains, the neomycin resistance (NeoR) cassette was amplified from pCAM-Neo regulated by TrpC promoter and TrpC terminator. The GFP expression cassette was cloned into the plasmid to generate pCAMBIA1302-neo-GFP. Then, the GFP open reading frame (ORF) was replaced with the VdHP1 ORF to generate pCAMBIA1302-neo-VdHP1 for *C-ΔVdHP1* (69).

### Southern blotting.

Southern hybridization was performed using a DIGHigh prime DNA labeling and detection starter kit II (Roche, Germany) according to the manufacturer’s protocol. Fragment hyg (selective marker gene, [798 bp]) was amplified for use as probe. DNAs of WT, *ΔVdHP1-1*, and *ΔVdHP1-2* mutants were digested by HindIII. The primers used in this assay are listed in Table S1.

### The morphology of mutation conidia.

The conidia derived from Vd080, *ΔVdHP1*, and *C-ΔVdHP1* strains were immersed in Glutaric dialdehyde, and stored overnight at 4°C. Samples were washed in 1 × PBS buffer (Solarbio, pH 7.2–7.4), and washed and dehydrated in a graded ethanol series 30%, 50%, 75%, 95%, 100% with critical point dried. Dried samples were observed via scanning electron micrographs (Hitachi SU-3000, Japan).

### Growth of mutation on stress treatments.

To test strains sensitivity to the cell wall inhibitors, osmotic stresses, and UV stress, all strains were cultured in PDA containing 0.4 M NaCl, 0.8 M KCl, 1.2 M sorbitol, 50 mg/mL CR or treated with a 10-s pulse of 302 nm UV light (18, 52, 69). The formula of relative growth inhibition rate was calculated as follows: relative growth inhibition rate = (control colony diameter – treatment colony diameter)/control colony diameter ×100% (12, 70). The mycelial growth of WT (the diameter of colony: 54.185 mm) at 25°C without any stress agents was the control. Each experiment was repeated three times.

For the thermostability assay, the conidia (1 × 10^7^ CFU/mL) of all strains were collected from 5 days Czapek liquid medium. An aliquot of 50 μL suspensions was heated at 45°C for 1 h, and then the conidia were spread on PDA for 22 h at 25°C (13). Germination was observed microscopically and the percent germination was recorded via examination of at least 100 conidia. Each experiment was repeated three times.

### Carbon source utilization assays.

To analyze carbon source utilization of WT, *ΔVdHP1* and *C-ΔVdHP1*, skim milk (18 g/L), cellulose (5 g/L), and starch (1 g/L) were individually added to Czapek Dox medium lacking sucrose. The experiment was repeated three times.

### Hydrophobicity.

The hydrophobicity of hyphal surface of colonies was tested by dropping 20 μL of 0.5% aqueous aniline blue on fully grown (6 d) colonies of WT, *ΔVdHP1-1*, *ΔVdHP1-2*, *C-ΔVdHP1-1*, and *C-ΔVdHP1-2*. Then, we observed and recorded the disappearance of the water or dye over a 45-min period (50, 71). The experiment was repeated three times.

Conidia of WT, *ΔVdHP1*, and C-*ΔVdHP1* strains were collected, respectively, and re-suspended in sterile water, 0.1% Tween 20, vortexed for 3 min, then ultrasonically treated for 2 min. The obtained spores were observed under a light microscope (72).

Microbial adhesion to hydrocarbons assay can determine cell surface hydrophobicity (34). Conidia harvested at 5-days-old in Czapek liquid medium were washed into PBS buffer. Fungal cell suspensions were adjusted to an OD450 = 0.3 and dispensed (1 mL) into 2.0 centrifuge tubes. N-hexane (300 μL) was then added to each tube. The tubes were vortexed for 2 min and then left to stand at room temperature for 15 min. The A450 of the resultant cell suspensions were determined. The hydrophobic index was calculated using the following equation: (A450_control_ − A450_n-hexane_)/A450_control_ (73).

### Mycelial penetration assays.

The conidia were spread onto the cellulose membrane (φ = 0.45 μm) or cellophane to observe effect on microsclerotia and the difference of mycelial penetration of different strains. The image of the microsclerotia was taken under a stereomicroscope (Leica M165C). The hyphae onto the cellophane were imaged by using the scanning electron microscopy. The experiment was repeated three times.

### Pathogenicity assays.

All strains (WT, *ΔVdHP1*, and *C-ΔVdHP1* strains) were incubated in liquid Czapek Dox medium (2 g/L NaNO_3_, 0.5 g/L KCl, 0.02 g/L FeSO_4_·7H_2_O, 0.5 g/L MgSO_4_·7H_2_O, 30 g/L sucrose and separately sterilized 1.31 g/L K_2_HPO_4_) at 25°C, 150 rpm for 5 days, and then diluted to 1 × 10^7^ CFU/mL, and take 5 μL for follow-up experiments.

*Gossypium hirsutum* cultivar “Jimian11” was used as host to perform pathogenicity assays. When the first euphylla was fully expanded, the cotton seedlings were inoculated with conidial suspension (1 × 10^7^ CFU/mL) for 15 min by the unimpaired root-dip inoculation method (16, 74). Ten to 25 days after inoculation, the disease was investigated many times. Three individual experiments were carried out for each strain, and repeated at three times. According to the symptoms observed from cotyledons and true leaves, the seedlings were divided into five grades (0, 1, 2, 3, or 4). The assessment of disease was conducted in cotton (75). For cotton plants, the DI was calculated according to the following formula (76): DI = [(Σ disease grade × number of infected plants)/(total number of sampled plants × 4)] × 100.

### Fungi recovery assay.

To confirm the ability of strains to approach the vascular system in cotton, the cotton stem 4 cm to 5 cm from the soil was collected at 21 dpi. The stem of the same part was cut into segments and cleaned with 75% ethanol and sterile distilled water, and then placed on PDA with kanamycin. The fungal recovery experiment was carried out at 25°C for 7 days to observe V. dahliae colonies. The assay was repeated three times.

### Confocal observation of the infection process.

Seeds of the cotton cultivar “Jimian11” were placed in a seed germination bag. After 7 days, cotton seedlings with uniform size were immersed in *ΔVdHP1-GFP-1* or *ΔVdHP1-GFP-2* or Vd080-GFP (stored in our laboratory) conidial suspension (1 × 10^7^ CFU/mL) for 10 min. Hyphal development in roots of cotton after inoculation was assessed at 2 dpi using a confocal laser scanning microscope.

After being washed with sterile water, onion epidermal (kept in 75% ethanol) were placed on water agar (10 g/L agar) plates. Conidial suspensions drops of 5 μL were loaded on the surface, and the plates were incubated at 25°C for 24 hpi, 48 hpi, 3 dpi. before examination under light microscope.

### Determination of crude toxin.

The spore suspension was cultured in liquid Czapek Dox medium for 10 days, under dark, 25°C, 180 rpm. After centrifugation, the supernatant of spore suspension was filtered through a 0.45-mm Millipore filter and used for crude toxin extract following the method (23). According to the Coomassie brilliant blue Gmur250 method, the standard curve was made under the spectrophotometer, and the protein concentration was determined.

### DNA extraction and expression analysis.

To detect the fungal biomass of cotton stem, total DNA of plants was extracted using Fungal DNA kit (Omega Bio-tek, Norcross, USA) from cotton stems 21 dpi. The cotton *Act* gene was used as the internal reference, and the specific *Vdβt* gene was used as the target sequence for detecting V. dahliae. All primers used in the assays are listed in Table S1. The relative content of all samples was calculated by 2 ^-ΔΔCT^ method (77–79). Three independent biological and technical repeats were performed.

### Statistical analysis.

In this study, three independent repeated experiments were carried out. A nested analysis of variance (ANOVA) was performed by Statistix 8.1 software using the mixed effect model.
