# Supplementary material for: Identification and Functional Analysis of a Novel Hydrophobic Protein VdHP1 from Verticillium dahliae
Source: Microbiol Spectr. 2022 Apr 4;10(2):e02478-21. doi: 10.1128/spectrum.02478-21 (PMC9045179; doi:10.1128/spectrum.02478-21)
Supplement: SUPPLEMENTAL FILE 1 — Supplemental material. Download SPECTRUM02478-21_Supp_1_seq3.pdf, PDF file, 0.7 MB [file spectrum02478-21_supp_1_seq3.pdf]

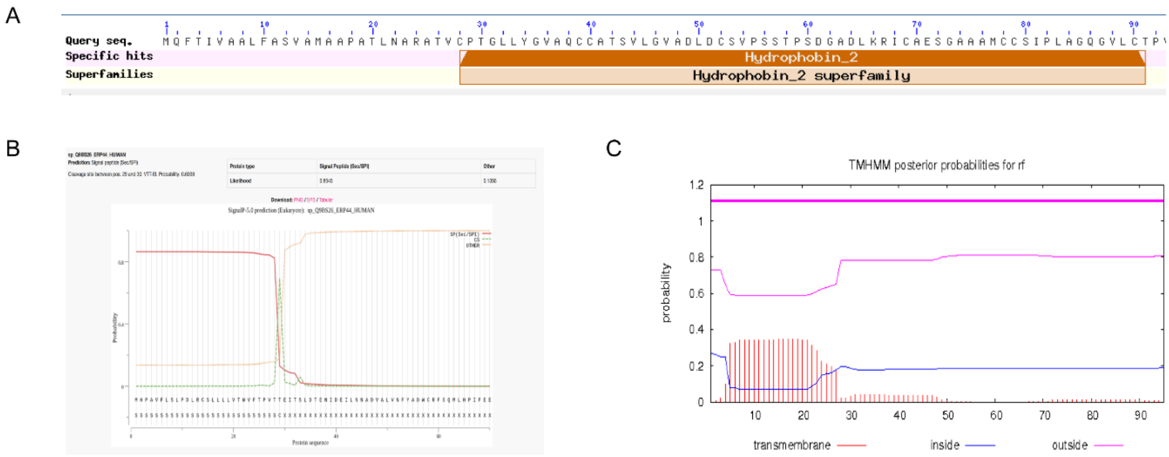

FIG S1: Bioinformatics analysis of VdHP1. (A) The conserved domain of VdHP1. (B) Transmembrane domain analysis of VdHP1. (C) The signal peptide sites of VdHP1 protein.

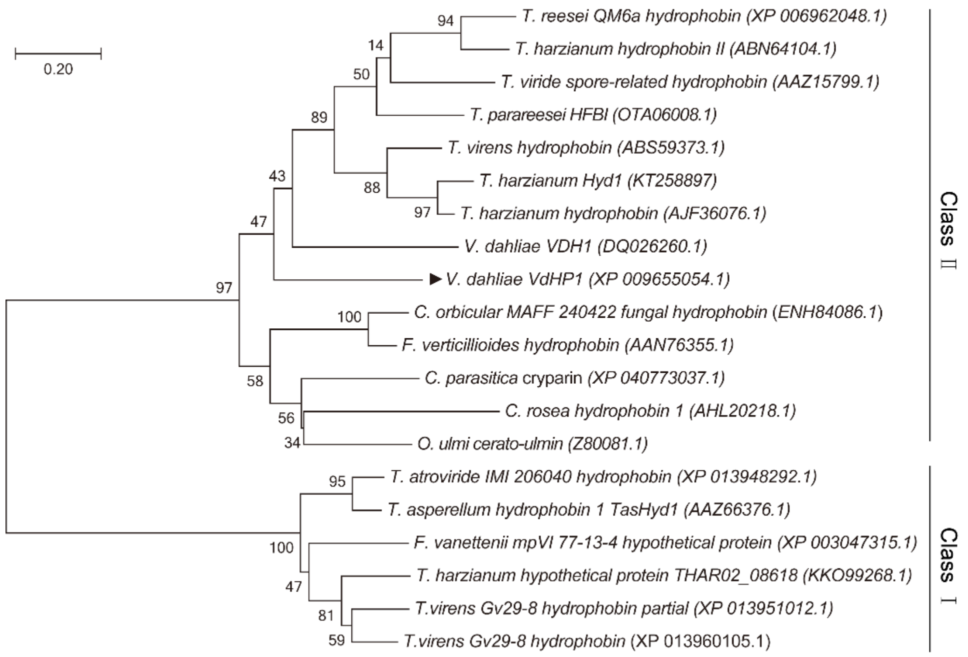

FIG S2： Phylogenetic analysis. MEGA 7 software used to construct phylogenetic neighbor-joining tree of the VdHP1 protein sequences and some hydrophobins sequences. The VdHP1 protein was marked with a black triangle and the hydrophobins sequences are from the GenBank database.

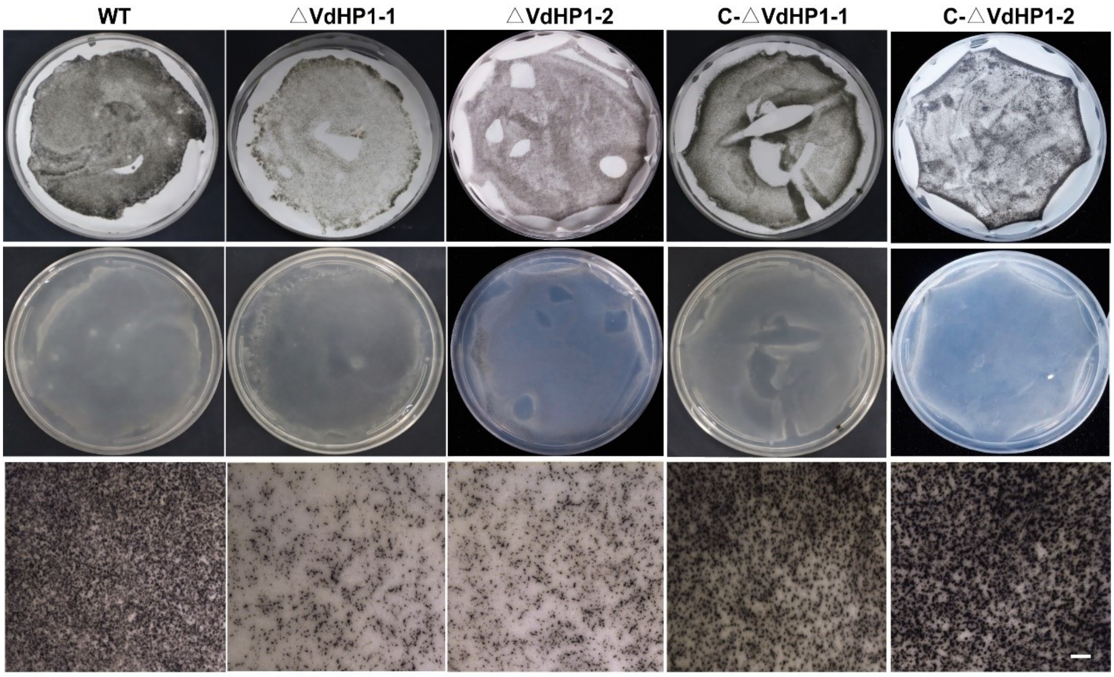

FIG S3: Deletion of VdHP1 inhibited microsclerotial formation. The conidia were spread over the cellulose membrane (φ = 0.45 μm) after 6 dpi of growth. The image of the microsclerotia was taken under a stereomicroscope. Bar = 5 mm

Supplementary Table 1：Primers used in this study.

Primer name	Sequence (5’-3’)	Enzyme loci	In this study
VdHP1-F	ATGCAGTTCACCATCGTCG		PCR,qPCR,sqRT-PCR
VdHP1-R	TTAACCAACGACGGGAGTGC		PCR,qPCR,sqRT-PCR
Hyg-F	GACGTTAACTGATATTGAAG		PCR,qPCR
Hyg-R	CGTCCGAGGGCAAAGGAATAG		PCR,qPCR
B303-VdHP1-UP-F	GGACCGGACGGGGCGGTACCGCGCTGACGACATGCCTT	KpnI	deletion of *VdHP1*
B303-VdHP1-UP-R	TTTGAAGGTGGTTTGGTTTGGTTGGTGGTGGTGGTAGAGGTTG		deletion of *VdHP1*
B303-VdHP1-Hyg-F	CAAACCAAACCACCTTCAAAGACGTTAACTGATATTGAAG		deletion of VdHP1
B303-VdHP1-Hyg-R	ACACGATTGAGTCAGAACTTCGTCCGAGGGCAAAGGAATAG		deletion of VdHP1
B303-VdHP1-DOWN-F	CTATTCCTTTGCCCTCGGACAAG TTCTGACTCAATCGTGTGAT		deletion of VdHP1
B303-VdHP1-DOWN-R	TTAATTAAGACCCGGGACTAAAGCACATCTCTCCAGCAGC	PacI	deletion of VdHP1
1302 -VdHP1-UP-F	GACAGCCCGCAAACTCTAGAGCGCTGACGACATGCCTT	XbaI	complementation of VdHP1
1302 -VdHP1-UP-R	GCGACGATGGTGAACTGCATTTTGAAGGTGGTTTGGTTTGG		complementation of VdHP1
1302-VdHP1-F	CCAAACCAAACCACCTTCAAAA TGCAGTTCACCATCGTCGC		complementation of VdHP1
1302-VdHP1-R	ACACGATTGAGTCAGAACTTTTA ACCAACGACGGGAGTGC		complementation of VdHP1
1302-VdHP1-DOWN -F	AAGTTCTGACTCAATCGTGTGAT TTGTATGCGGCGCAATTCT		complementation of VdHP1
1302-VdHP1-DOWN -R	ATTATTATGGAGAAACTCGAGAAGCACATCTCTCCAGCAGC	XhoI	complementation of VdHP1

Vdβt-F	AACAACAGTCCGATGGATAATTC		qRT-PCR
Vdβt-R	GTACCGGGCTCGAGATCG		qRT-PCR
Act-F	CCTATGTTGCCCTGGACTATGAGC		qRT-PCR
Act-R	GGACAACGGAATCTCTCAGCTCC		qRT-PCR
Hyg probe-F	atttgtgtacgcccgacagt		for Southern blotting
Hyg probe-R	gtgcttgacattggggaatt		for Southern blotting
pSUC2-VdHP1-F	CGGAATTCATGCAGTTCACCATCGTCGC	EcoR1	Yeast signal sequence trap system
pSUC2-VdHP1-R	CCGCTCGAGGGGCAGACGGTGGCA	Xho1	Yeast signal sequence trap system
NbActin-F	TGGTCGTACCACCGGTATTGTGTT		sqRT
NbActin-R	TCACTTGCCCATCAGGAAGCTCAT		sqRT
